# Pediatric Thyroid Lesions: Synergistic Role of Clinical and Cytological Features in Diagnosis

**DOI:** 10.15190/d.2023.3

**Published:** 2023-03-31

**Authors:** Anurag Singh, Pallavi Prasad, Alka Singh

**Affiliations:** ^1 ^Department of Pathology, Sanjay Gandhi Postgraduate Institute of Medical Sciences, Lucknow, India

**Keywords:** Fine-needle aspiration, cytology, histopathology, pediatric, thyroid function test, ultrasonography.

## Abstract

INTRODUCTION: Thyroid lesions in childhood and adolescence are uncommon, and the risk of malignancy widely varies. They require careful evaluation and more aggressive diagnostic approach. The present study aimed to evaluate the frequency of various pediatric thyroid lesions in pediatric cases with thyroid nodules and ascertain the utility of clinical, laboratory, ultrasonography, and fine-needle aspiration cytology (FNAC) findings to discriminate between benign and malignant lesions. METHODS: A retrospective study where 95 consecutive cases of pediatric patients with thyroid nodules received over six years (January 2016–December 2021) were retrieved from the hospital information system. The differences in clinical, laboratory, ultrasonography, and cytological findings between benign and malignant lesions were analysed. Statistical analysis was performed using SPSS software (version 21.0). RESULTS: The Bethesda System for Reporting Thyroid Cytopathology (TBSRTC) was used to categorise the cases into: unsatisfactory (n=3), benign (n=66), intermediate (n=8) and suspicious/malignant (n=18). The specificity of cytopathology in diagnosing benign lesions (TBSRTC-II) was 90%, whereas sensitivity in diagnosing malignant lesions (TBSRTC-VI) was 100%. Colloid nodule (n=57) and papillary thyroid carcinoma (n=15) were the most common benign and malignant lesions encountered respectively. Malignant lesions more frequently showed the presence of palpable lymph nodes (p-value <0.001), microcalcifications (p-value 0.011) and intranodular vascularization (p-value <0.001). CONCLUSION: The diagnosis of pediatric thyroid lesions should be based on a multistep evaluation that includes clinical, laboratory, and radiographic modalities. Thyroid function tests and ultrasonography can help identify clinically unapparent thyroid nodules and provide detailed nodule characterization for suspected malignant lesions. FNAC is a simple, less-invasive, and cost-effective technique that can differentiate between benign and malignant thyroid lesions.

## INTRODUCTION

Thyroid lesions are uncommon in children and adolescents, with an estimated prevalence of 0.05% to 1.8%. The reported incidence in the adult population is relatively higher, ranging from 3.2% to 8%^1^. Risk factors for development of thyroid nodules in children may include head and neck irradiation, female gender, iodine deficiency, age of puberty, and family or personal history of thyroid disease^2^.The probability of malignant transformation in children varies considerably, ranging from 5% to 50% in different studies, while it is comparatively lower in adults varying from 5% to 15%. This necessitates a thorough assessment and a more aggressive diagnostic strategy in children^3-5^. The most frequent endocrine malignancy in children is thyroid carcinoma^6-8^.When compared to their adult counterparts, pediatric thyroid malignancies more frequently present with advanced disease, including lymph node involvement and the presence of distant metastasis^4,9-11^.A variety of techniques including ultrasonography, radionuclide scans, fine needle aspiration (FNA), and thyroid function test (TFT) are used to characterise a thyroid lesion. All these modalities have been extensively investigated in adults. However, because of the difficulty in collecting a larger sample size, this is generally more difficult and troublesome in children. The current study aimed to determine the prevalence of thyroid malignancies in children with thyroid nodules and study the significance of clinico-radiological and cytological findings in distinguishing between benign and malignant lesions. Another objective was to study the utility of The Bethesda System for Reporting Thyroid Cytopathology (TBSRTC) system in distinguishing between benign and malignant pediatric thyroid lesions.

## MATERIAL AND METHODS

A total of 95 cases of pediatric thyroid nodules were studied retrospectively over a period of six years (January 2016-December 2021) at Sanjay Gandhi Postgraduate Institute of Medical Sciences, Lucknow. All subjects below 18 years of age who underwent FNA for thyroid lesions at our institute were included. Cases with a history of oncohematological illness or prior radiation exposure were excluded from the study. The hospital information system and medical record files were used to retrieve clinical, laboratory, radiographic, cytological, and histopathological data. The patient's age, sex, status of palpable thyroid nodule and lymph nodes were documented. Highly specific fluorometric or enzyme-linked immunoassays were used to assess the thyroid function tests: thyroid-stimulating hormone (TSH), and free thyroxine (FT4) levels for these patients. Thyroid ultrasonography was performed on all patients to determine the number of nodules, maximum diameter of the largest thyroid nodule, echoic pattern, consistency, presence of microcalcification, and vascularity. A 23-gauze needle was used to perform the FNA, which was hooked to a disposable 10 mL or 20 mL syringe with or without a holder. Ultrasonographic assistance was used to aspirate tiny nodules, and the aspirated material was spread onto glass slides. The smears were stained with May-Grünwald-Giemsa (MGG), hematoxylin and eosin, and Papanicolaou (Pap) stains. All cytology smears were examined and classified using the TBSRTC. In 57 individuals who underwent surgery, histological follow-up was also available. All the data was collected, compiled, and evaluated systematically to compare the findings between benign and malignant lesions and to assess the sensitivity and specificity of FNAC in different categories of TBSRTC.

Statistical data were analysed using the Statistical Package for Social Sciences (SPSS; IBM Inc., USA) version 21.0. The Chi-square test was used for the comparison of categorical variables, and independent t-test was used for the analysis of parametric data. A p-value less than 0.05 was considered statistically significant.

## RESULTS

The mean age at diagnosis for pediatric thyroid lesions was 15.58+2.49 years and were more common in girls (63.16 %) compared to boys (36.84 %). When comparing benign and malignant lesions, neither the age (p-value = 0.075) nor the gender (p-value = 0.814) of the cases showed a significant difference (Table 1). TBSRTC was used to assess the cytology smears for consistent reporting of thyroid nodules. This six-tier approach aided in the differentiation of benign, intermediate and malignant thyroid lesions. On FNAC, 69.47% of cases (66/95) were negative for malignancy. Among them, colloid nodule (57/66) was the commonest diagnosis. Histology was available in 45.45% of benign lesions (30/66) reported on cytology, of which it was in concordance with the cytological diagnosis in 27 of 30 cases. Three cases reported as benign in cytology turned out to be malignant (2 follicular carcinoma and 1 papillary thyroid carcinoma). Histology was available in all the 3 cases of follicular lesion of undetermined significance/atypia of undetermined significance (FLUS/AUS) category (3/3); 2 cases were diagnosed as multinodular goitre (MNG) and one as colloid nodule (CN).

**Table 1:** Clinical and ultrasonographic features in benign and malignant lesions of the thyroid

	Total (n=95)	Total (n=95) %	Benign (n=72)	Benign (n=72) %	Malignant (n=23)	Malignant (n=23) %	p-value
Age (mean± SD)	15.59 ± 2.49		15.85 ± 2.16		14.78 ± 3.26		0.075
Gender							
Male (n, %)	35	36.84	27	37.50	8	34.78	0.814
Female (n, %)	60	63.16	45	62.50	15	65.22	
Thyroid function test							
Euthyroid (n,%)	80	84.21	58	80.56	22	95.65	0.192
Hyperthyroid (n,%)	7	7.37	7	9.72	0	0.00	
Hypothyroid (n,%)	8	8.42	7	9.72	1	4.35	
Ultrasonographic findings							
Maximum nodular diameter (cm) (mean± SD)	3.72±1.58		3.77±1.34		3.57±2.14		0.606
Consistency of lesion							
Cystic (n,%)	16	16.84	15	20.83	1	4.35	0.182
Solid (n,%)	42	44.21	30	41.67	12	52.17	
Solid-cystic (n,%)	37	38.95	27	37.50	10	43.48	
Pattern of echogenicity							
Anechoic (n,%)	10	10.53	9	12.50	1	4.35	0.056
Hypoechoic (n,%)	22	23.16	13	18.06	9	39.13	
Hyperechoic (n,%)	18	18.95	17	23.61	1	4.35	
Isoechoic (n,%)	17	17.89	14	19.44	3	13.04	
Mixed echogenicity (n,%)	28	29.47	19	26.39	9	39.13	
Increased vascularity (n,%)	23	24.21	4	5.56	19	82.61	<0.001
Microalcification of nodule (n,%)	18	18.95	9	12.50	9	39.13	0.011
Palpable thyroid nodule (n,%)	81	85.26	63	87.50	18	78.26	0.453
Palpable lymph nodes	21	22.11	6	8.33	15	65.22	<0.001

**Figure 1:**
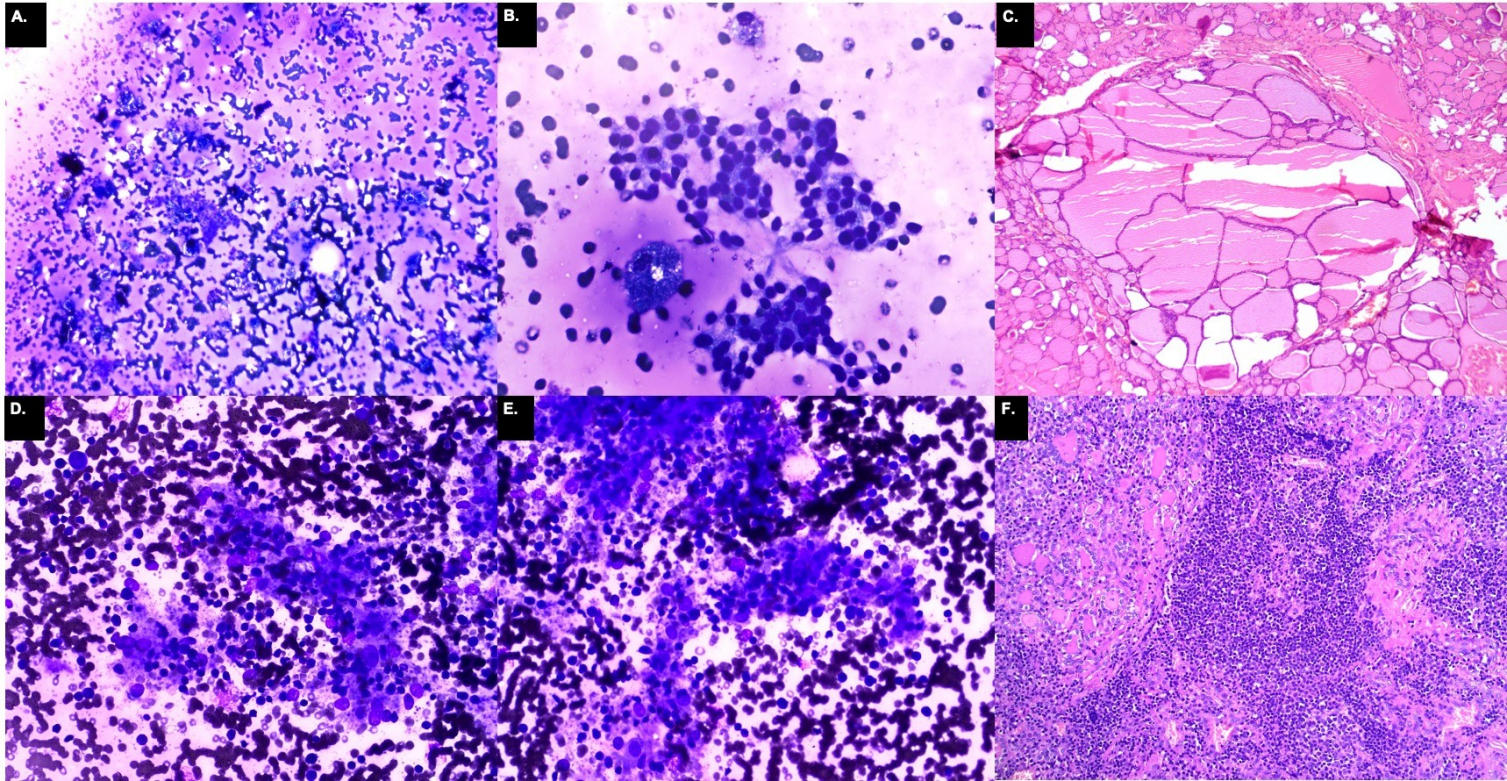
Case of colloid nodule showing follicular epithelial cell clusters and cystic macrophages on a background of abundant colloid (A. MGG x100, B. MGG x200), confirmed on histopathology (C. H&E x100). Case of lymphocytic thyroiditis showing impingement of lymphocytes over follicular epithelial cells (D. MGGx100, E.MGGx200, F. H&E x100).

**Figure 2:**
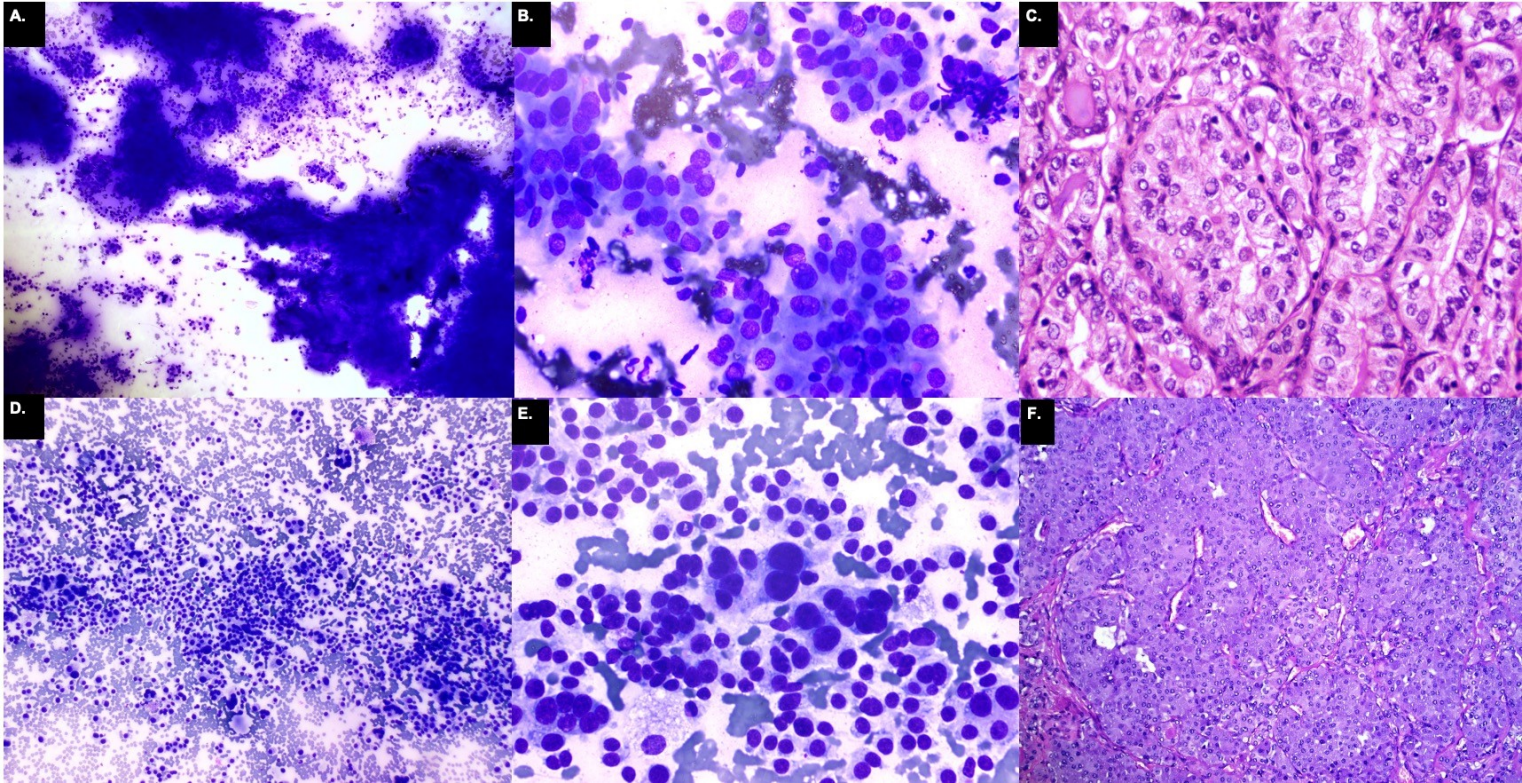
Papillary thyroid carcinoma showing papillaroid fronds with central fibrovascular cores (A. MGG x100), pseudoinclusions and occasional grooving (B. MGG x400), confirmed on histopathology (C. H&E x400). Medullary thyroid carcinoma showing a dispersed population of plasmacytoid cells (D. MGG x100, E. MGG x400), confirmed on histopathology (F. H&E x200).

**Figure 3:**
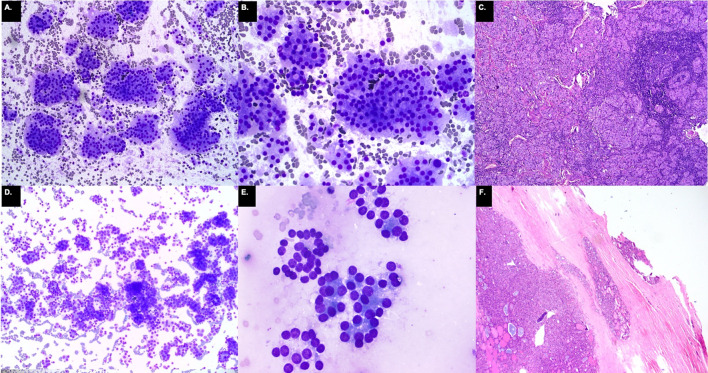
Cytology images showing an exclusive population of hurthle cells (A. MGG x100, B. MGG x200), confirmed on histopathology as hurthle cell nodule (C. H&E x100). Cytology images showing repetitive microfollicular pattern (A. MGG x100, B. MGG x400), confirmed on histopathology as follicular carcinoma (c. H&E x100).

**Figure 4:**
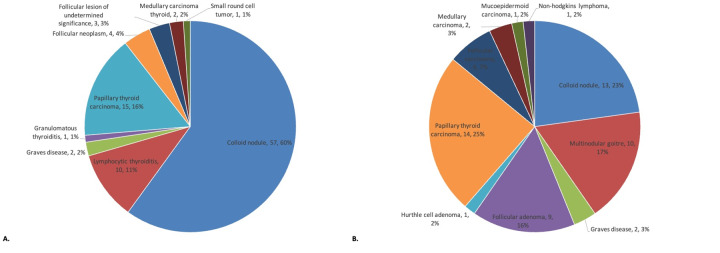
Distribution of cases based on (A.) cytology and (B.) histology.

Figure 1, Figure 2 and Figure 3 show images of representative cases. The distribution of cases on cytology is given in Figure 4A. With 100% sensitivity and specificity, all FNACs with the diagnosis of suspicious or positive for malignancy (17) were diagnosed as malignant on subsequent histology (17/17). 3 cases reported as non-diagnostic/unsatisfactory on FNAC were diagnosed as colloid nodule on histology (Table 2).

**Table 2:** Correlation of cytological and histological diagnosis *In the cases confirmed as malignant on histopathology (n=16), all were identified malignant by cytopathology (sensitivity of cytopathology: 100%). In the cases diagnosed as benign by cytopathology (n=30), 27 were confirmed as benign on histopathology (specificity of cytopathology: 90%). **AUS- atypia of undetermined significance, FLUS-follicular lesion of undetermined significance, FN-follicular neoplasm, SFN-suspicious for follicular neoplasm, CN-colloid nodule, MNG- multinodular goitre, GD- grave's disease, FA-follicular adenoma, FC-follicular carcinoma, PTC-papillary thyorid carcinoma, HCA-hurthle cell adenoma, MTC-medullary carcinoma, MEC-mucoepidermoid carcinoma, NHL-non-hodgkin's lymphoma.

TBSRTC category	Cytological Diagnosis	Cytological Diagnosis	Histological diagnosis	Number of cases diagnosed as malignant (%)
	N=95	%	N=57	N=22/57 (38.6%)
I (non-diagnostic/ unsatisfactory)	3	3.15%	03 (5.26%)	0 (0%)
			CN (03)	
II (Benign)	66	69.47%	30 (52.6%)	3/30 (10%)
			CN (09)	
			MNG(8)	
			GD (2)	
			FA (8)	
			FC (2)	
			PTC (1)	
III (AUS)/FLUS	3	3.15%	3 (5.26%)	0 /3(0%)
			CN (1)	
			MNG (2)	
IV (FN/SFN)	5	5.26%	4 (8.77%)	2/4 (50%)
			HCA (1)	
			FA (1)	
			FC (2)	
V (Suspicious for malignancy)	1	1.05%	1 (1.75%)	1/1 (100%)
			PTC (1)	
VI (Malignant)	17	17.89%	16 (28.07%)	16/16 (100%)
			PTC(12)	
			MC (2)	
			MEC (1)	
			NHL (1)	

The histology was available in 57 cases, of which 22 and 35 cases were reported as malignant (22/57) and benign (35/57) respectively. The distribution of cases on histology is given in Figure 4B. Among the benign ones, the most common diagnosis was colloid nodule (CN) (13/35), followed by multinodular goitre (MNG) (10/35), follicular adenoma (FA) (9/35), grave's disease (2/35) and hurthle cell adenoma (1/35). The malignant cases comprised papillary thyroid carcinoma (14/22), follicular carcinoma (4/22), medullary carcinoma (2/22), primary non-Hodgkin's lymphoma (1/22), and mucoepidermoid carcinoma (1/22). A total of eight cases were reported as FLUS/AUS and FN/SFN on cytology, and histology was available in seven cases, in which they were diagnosed as follicular carcinoma (2/7), MNG (2/7), hurthle cell adenoma (1/7), follicular adenoma (1/7) and colloid nodule (1/7). Hence, the specificity of cytopathology in diagnosing benign lesions (TBSRTC-II) was 90%, whereas sensitivity in diagnosing malignant lesions (TBSRTC-VI) was 100% (Table 2).

The clinical, biochemical, and ultrasonographic features of individuals with benign and malignant thyroid lesions are compared in Table 1. A significant difference was noted in the clinical characteristics and ultrasonographic findings between benign and malignant lesions. Malignant neoplasms were more likely to present with palpable lymph nodes (65.22 % vs. 8.33 %; p-value <0.001), microcalcifications (39.13 % vs 12.50 %; p value 0.011) and intranodular central vascularization (82.61 % vs 5.56 %; p-value <0.001). However, no significant difference was noted in the consistency of thyroid nodule (p-value =0.182), maximum nodule diameter (p value 0.606), echoic pattern (p-value =0.056) and thyroid function test (p-value =0.192) (Table 2).

Serum calcitonin was estimated in 35 cases and found to be elevated in 2 of them (910 pg/mL and 315 pg/mL, respectively). Both patients underwent surgery and were diagnosed as medullary carcinoma on histology.

## DISCUSSION

The possibility of malignancy in thyroid swelling should be suspected in children and adolescents since they have a greater risk of malignancy than adults^3,12-16^. The incidence of malignancy in thyroid nodules of pediatric patients has been reported to range from as low as 9.2%^5^to as high as 50%^14^in different studies. Given the patient's age and degree of anxiety, FNA should be performed as a first-line investigation. Surgical excision should be undertaken for diagnosis in cases where FNAC smears are inadequate or in those in whom FNA cannot be performed. Total thyroidectomy should be reserved for cases that are preoperatively diagnosed as malignant, whereas lobectomy should be done in lesions where the cytologic diagnosis is equivocal^15,17-20^. Thyroid carcinoma has an exceptionally better prognosis in children, with a 10-year survival rateof approximately 98.8%^7,10^.

In this study, we report a cohort of pediatric patients with thyroid lesions that were identified using clinical, laboratory, and ultrasonographic evaluation. The higher prevalence of thyroid lesions seen in females in our study cohort has already been documented in the literature and is most likely due to estrogen sensitivity of the thyroid gland^21-24^.

We report a significant association between enhanced intranodular vascularity and microcalcification on ultrasound with malignancy, which is in concordance with previous studies^25,26^.However, no significant difference was noted in maximum nodule diameter or the consistency of nodules between benign and malignant lesions. Although the majority of malignancies (39.13%) had a hypoechoic pattern during the ultrasonographic assessment, this characteristic was of limited clinical usefulness, as it was frequently noted in benign lesions (18.06%) as well. Because the great majority of pediatric thyroid lesions (84.21%) and most malignant neoplasms (95.65%) develop in euthyroid state, thyroid function tests were of no use in distinguishing between benign and malignant cases. Hence, clinical and ultrasonographic examination should be the primary modalities in the diagnostic workup of a thyroid lesion, followed by FNAC.

TBSRTC offers a practical and reliable thyroid FNAC reporting system, and is the most accurate approach, according to the American Thyroid Association^27^. This approach has been used in several studies to report thyroid cytology in adults^28,29^.However, only a few studies have utilised this system to report pediatric thyroid FNAs^30-33^.TBSRTC presents a six-tier method for reporting thyroid FNAC uniformly, as well as assisting in the development of a consistent management plan and treatment guidelines. This study brings forth the efficacy of TBSRTC system in distinguishing between benign and malignant thyroid lesions in children. FNA is quite essential in preoperative planning. If the lesion is found to be malignant, the patient should undergo a complete thyroidectomy rather than a lobectomy. When the diagnosis is inconclusive or benign, an initial lobectomy is indicated. FNA cannot detect capsular or vascular invasion, hence, follicular lesions cannot be classified as malignant or benign. If the nodule is identified as benign by FNA, the patient can be observed while the lesion is small and operational intervention can be avoided. The accuracy of FNAC can reach up to 97% in adults, but it only reaches 90% in children^4,9,34-36^.Other limitations of FNAC include sample inaccuracy, the cytopathologist's expertise, and the requirement for anaesthesia in younger children.

Malignancy was noted in 23.15% of patients (22/95) in our study. Observations from previous studies state that malignant neoplasms are more common in pediatric thyroid lesions when compared to adults^3,5,14^. In the study, papillary thyroid carcinoma was the most common type of cancer. This was followed by follicular carcinoma, medullary carcinoma, mucoepidermoid carcinoma, and primary non-Hodgkin's lymphoma of the thyroid.

Because of the extensive use of neck ultrasonography and other imaging modalities in recent years, a large number of thyroid nodules in adults have been detected by chance^37^. Thyroid nodules are commonly accidentally diagnosed in children and adolescents. In the current study, 14.7% (14/95) patients were referred to an endocrinologist because of incidental detection of a thyroid nodule on ultrasonography, with 14.3% (2/14) cases identified as papillary thyroid carcinoma and the remaining 85.7% (12/14) as benign. There is considerable debate over whether clinically inapparent thyroid lesions should be assessed by FNAC because some authors attribute the same risk of malignancy to accidentally discovered thyroid nodules as to clinically evident nodules^27,38,39^ whereas others suggest that non-palpable thyroid nodules present an overall low risk^37^. In our series, the proportion of malignancy in patients with incidentally discovered nodules (14.3%) (2/14) was found to be similar to the rate of malignancy found in individuals with palpable nodules (19.7%) (16/81). As a result, it is appropriate to state that both palpable and non-palpable pediatric nodules pose the same concern, and a uniform diagnostic approach should be adopted.

The diagnostic accuracy of the FNAC was high (88.7%) and was only a few percentage points lower than that reported in earlier studies (90.4%)^26,36^. As a result, FNAC permits a limited number of patients to undergo surgery. Laboratory tests are of limited utility in the diagnosis and treatment of thyroid lesions. However, serum calcitonin level may be included as it aids in the early detection of medullary carcinoma^40^ as was observed in both patients in our study. Due to the rarity of thyroid lesions in the pediatric population, the data in this study was collected retrospectively; this was a major limitation of the study.Furthermore, because some of the patients in the study were deemed to have benign lesions solely based on clinical, ultrasonography, and FNAC results, our findings may understate the real frequency of malignant lesions in the pediatric population. Furthermore, statistics must be evaluated in light of the fact that India suffers from mild to moderate iodine insufficiency, which leads to an increased incidence of goitre and, as a result, the frequency of benign nodules may be overstated.

Our results indicate that malignant neoplasms were more likely to present with palpable lymph nodes (65.22% vs 8.33%; p-value<0.001), microcalcifications (39.13% vs 12.50%, p-value 0.011) and intranodular central vascularisation (82.61% vs 5.56%; p-value=0.001). However, no significant difference was noted in the consistency of thyroid nodule (p-value=0.182), maximum nodule diameter (p-value 0.250), echoic pattern (p-value=0.056) and thyroid function tests (p-value=0.192). Knowledge of these data would help avert surgery in selected pediatric cases with thyroid nodules.

## CONCLUSION

We recommend a well-thought-out combination strategy for the diagnosis of pediatric thyroid lesions that includes a thorough clinical examination and ultrasonography followed by FNAC. In most situations, thyroid lesions with palpable lymph nodes should alert clinicians since they are more likely to be associated with malignancy. Although ultrasonography cannot consistently distinguish between benign and malignant tumours, it offers an index of suspicion that aids in selecting individuals for FNAC. Malignancy is suspected when there are microcalcifications or enhanced intranodular vascularization. Ultrasonography can also detect nodules that aren't readily visible but could be malignant. FNAC is a cost-effective and safe technique, which because of its high diagnostic accuracy, helps in selecting patients for surgery.
